# Globalized parameter tuning of microwave passives by dimensionality-reduced surrogates and multi-fidelity simulations

**DOI:** 10.1038/s41598-025-05798-0

**Published:** 2025-07-01

**Authors:** Slawomir Koziel, Anna Pietrenko-Dabrowska

**Affiliations:** 1 Engineering Optimization & Modeling Center, Reykjavik University, 102 Reykjavik, Iceland; 2 Faculty of Electronics, Telecommunications and Informatics, Gdansk University of Technology, 80-233, Gdansk, Poland

**Keywords:** Computer-aided design; artificial intelligence-based optimization; global search, Sensitivity analysis, Surrogate modeling, Multi-fidelity computational models, Electrical and electronic engineering, Computational science

## Abstract

Parameter tuning is an essential but demanding aspect of microwave component design, particularly when global optimization is required. The process becomes especially demanding due to the extensive electromagnetic (EM) simulations involved, which—when using popular nature-inspired methods—can lead to unmanageable computational costs. Traditional mitigation approaches, such as surrogate-based methods, often struggle with the curse of dimensionality and the highly nonlinear responses of microwave circuits. This study introduces an alternative approach for rapid global optimization of microwave passive components using artificial intelligence (AI) techniques, specifically, machine learning (ML). The core elements of our methodology include reduction of the problem dimensionality using a rapid global sensitivity analysis, multi-fidelity EM simulations, and a two-stage search process. During the global optimization stage, surrogate-assisted ML is confined to a reduced-dimensionality region, leading to significant computational savings and enhanced predictive accuracy of the surrogate models. Additional speedup is achieved by performing the search using low-fidelity EM models. The final local refinement stage employs high-resolution models and is executed within the design space of full dimensionality, ensuring the quality of the final design. Our procedure was comprehensively validated using four microstrip circuits and has demonstrated superiority over state-of-the-art benchmark methods. The average optimization cost is equivalent to only about ninety EM simulations. Further, the quality of the resulting designs remains competitive with those rendered using the benchmark methods.

## Introduction

The structural sophistication of microwave passives has been increasing over the years due to performance demands pertaining to various application areas^1–6^. These are formulated either as requirements for electrical characteristics (broadband operation, harmonic suppression, multi-band operation^7–9^), or circuit functionality^10,11^. Following miniaturization trends, maintaining a small size has rendered a key consideration^12–15^, which can be achieved using, e.g., metamaterials^16,17^, transmission line foldings^18^, compact resonant cells^19^, or defected grounds^20^. Increasing geometrical complexity of passive components makes their design process intricate, with electromagnetic (EM)-based parameter adjustment becoming imperative^21^. It is because equivalent network models are insufficient, especially in the presence of cross-coupling effects. They may not correctly account for dielectric losses or the effects of interactions with diverse components (e.g., connectors).

While EM-based design is necessary^22–24^, it is also challenging. It requires handling multiple parameters, objectives, and constraints. Also, it entails considerable expenses due to massive EM analyses involved. Even local parameter adjustment may require from several dozen to a few hundred simulations. As a result, traditional interactive approaches (e.g., parametric studies) are often defaulted to, although they can only lead to obtaining inferior outcome and are arduous due to engaging human experts. Meanwhile, global optimization is fostered in various antenna development scenarios, e.g., problems featuring multiple local optima, development of metamaterials^25^, coding metasurfaces^26^, frequency selective surfaces^27,28^, pattern synthesis^29–32^. Further, it might be required if a good starting point for a local search is hard to obtain, e.g., while re-designing coupling or filtering circuits over broad ranges of frequency or material parameters^33,34^, or optimization of miniaturized structures^35^.

Nowadays, predominant global optimization methods are undoubtedly population-based bio-inspired procedures^36–40^. These techniques capitalize on mimicking social or natural phenomena^41,42^ (e.g., biological evolution^43,44^ feeding tactics of living organisms^44^). Nature-inspired algorithms process ensembles (populations, packs)^46^ of candidate solutions^47^. Exchanging data between the population members^48,49^, as well as the incorporation of stochastic components (randomized selection^50^, random mutation^51^, etc.) are the two factors that arguably enable the identification of global optimum. The procedures of this sort come in many variants. The most recognized approaches are evolutionary^52,53^ or genetic algorithms^54^, particle swarm optimizers (PSO)^55^, differential evolution (DE)^56^, ant or firefly algorithms^57,58^, and many others^59–64^. Population-based methods are simple to implement; however, their cost efficiency is inferior. Direct bio-inspired optimization of high-frequency structures is prohibitive in most cases provided that the circuit is characterized using EM analysis (unless the simulation time does not exceed ten to thirty seconds or so). Consequently, such algorithms are normally employed to optimize models described with the use of analytical functions (e.g., pattern synthesis of antenna arrays^65,66^).

Mitigating the cost-related issues can be achieved through surrogate modeling methods^67–69^. In practice, it is often realized using machine learning (ML)^70–72^, where a behavioral model (Gaussian process regression^73,74^, neural networks^75,76^) serves to find the optimum. The metamodel is iteratively improved using the collected EM-generated samples^77^. Generating new candidate solutions can be based on model accuracy improvement^78^ or optimum identification^79^. While ML may be efficient, its major vulnerability is to construct the metamodel itself. This task is hampered by the curse of dimensionality and the sheer size of the parameter space. Consequently, many ML frameworks have been illustrated using low-dimensionality cases^80–82^. The modeling process can be facilitated using methods adhering to performance-driven approach^83–86^, multi-resolution frameworks^87–89^, response feature technology^90–93^, and physics-based routines (e.g., space mapping, cognition-driven design^94–97^). Still, the mentioned are not sufficiently generic, e.g., feature-based optimization are contingent upon the existence the features across the entire parameter space^98^; space mapping relies on the appropriately defined low-resolution models. These are problem-dependent and generally not transferable between problem domains.

This paper introduces an innovative procedure for efficient global parameter tuning of microwave circuits leveraging artificial intelligence (AI) methods, specifically surrogate-assisted machine learning. Our framework incorporates data-driven surrogates and PSO, which act as the core optimizer. The cost-efficacy of the search procedure is improved by restricting it to a dimensionality-reduced affine subspace determined using the vectors that affect variability of the circuit responses in a most significant manner. The latter are determined using a rapid global sensitivity analysis. For additional acceleration, a global search is executed using a low-resolution EM model. At the same time, reliability is ensured by supplementary fine-tuning realized using gradient-based optimization at the high-resolution modeling level. Numerical verification of our methodology involves four microstrip circuits and comprehensive benchmarking against several techniques (nature-inspired optimization realized with the use of EM simulations, gradient-based optimization, ML operating within unreduced space, and dimensionality-constrained ML using EM simulations of high accuracy). The results corroborate the competitive efficacy of the proposed framework, especially regarding design quality and computational efficiency. The mean algorithm cost amounts to just ninety full-wave simulations at high-resolution level, comparable to typical expenses incurred by local parameter tuning.

## Variable-resolution microwave optimization by dimensionality-reduced surrogates and artificial intelligence-based methods

This section discusses the search algorithm suggested in this research. The formulation of the optimization problem (Section "EM-driven design of microwave circuits") precedes a discussion of multi-fidelity EM models (Section "Variable-fidelity modeling"). Section "Cost-effective global sensitivity analysis" outlines rapid global sensitivity analysis (RGSA) and a procedure for reducing the problem, whereas Section "Optimization process: AI-based global search phase" describes surrogate-assisted global search using machine learning. Sections "Optimization process: local parameter tuning" and "Optimization framework complete procedure" provide the particulars of a local parameter refinement and the entire optimization system, respectively. The optimization framework proposed in this study employs several AI tools, including kriging interpolation surrogates and the iterative ML scheme, where the surrogate is employed as a fast predictor and refined using the accumulated EM simulation data. Furthermore, a bio-inspired method (namely, PSO) is used to generate a candidate solution to the optimization task.

### EM-driven design of microwave circuits

Simulation-driven design is the adjustment of the circuit decision variables (here, geometry parameters) directed towards minimizing a scalar objective function *U*(***x***), measuring the quality of designs. Here, ***x*** is a vector of parameters over a search space *X* (see Table 1 for a summary of the notation). The circuit outputs (complex *S*-parameters vs frequency) are modelled via EM simulations. The design problem is stated as

1 $${\boldsymbol{x}}^{*} = \arg \mathop {\min }\limits_{{{\boldsymbol{x}} \in X}} U({\boldsymbol{x}})$$

**Table1:** Simulation-driven design of microwave devices: important notation.

Symbol	Explanation
***x*** = [*x*_1_ … *x*_*n*_]^*T*^	Designable parameters (usually circuit dimensions)
*S*_*ij*_(***x***,*f*)	Scattering parameters evaluated at the design ***x*** and frequency *f* (where *i* and *j* represent the circuit ports)
*X* = [***l u***]	Design space (typically box-constrained delimited using lower and upper bounds on parameters ***l*** = [*l*_1_ … *l*_*n*_]^*T*^, and ***u*** = [*u*_1_ … *u*_*n*_]^*T*^)
*U*(***x***)	Objective (cost) function assessing the design quality
*g*_*k*_(***x***) ≤ 0, *k* = 1, …, *n*_*g*_	Inequality constraints (typically, defined by imposing lower or upper acceptance thresholds for specific circuit responses over selected frequency ranges)
*h*_*k*_(***x***) = 0, *k* = 1, …, *n*_*h*_	Equality constraints (typically, constraints defined by imposing specific target values for selected operating figures, e.g., resonant frequency, of the circuit)

Here, x^*^ is the optimum we seek for. If multiple objectives are present, they are typically cast into constraints^99^ or aggregated^100^. Multi-objective design^101^ is beyond the interest of this research. In some cases, the problem (1) may be complemented by inequality and/or equality conditions, *g*_*k*_(***x***) ≤ 0 or *h*_*k*_(***x***) = 0 (cf. Table 1). In the case of expensive constraints, their convenient handling can be realized through penalty functions^99^, where the design task needs to be reformulated as

2 $${\boldsymbol{x}}^{*} = \arg \mathop {\min }\limits_{{\boldsymbol{x}}} U_{P} ({\boldsymbol{x}})$$

where *U*_*P*_ incorporates the original objective function *U* and the penalty factors

3 $$U_{P} ({\boldsymbol{x}}) = U({\boldsymbol{x}}) + \sum\nolimits_{k = 1}^{{n_{g} + n_{h} }} {\beta_{k} c_{k} ({\boldsymbol{x}})}$$

Observe that the penalty functions *c*_*k*_(***x***) in (3) quantify violations of the constraints (cf. Table 2), whereas coefficients *β*_*k*_ control the contribution of particular penalty terms. For the sake of illustration, Table 2 lists several specific examples of microwave optimization problem formulations. The constraints are treated implicitly, according to (2) and (3).

**Table 2:** Common design tasks in simulation-driven microwave design.

Design task	Objective function*
Circuit: Microstrip coupler	$$\begin{gathered} U({\boldsymbol{x}}) = \max \left\{ {|S_{11} ({\boldsymbol{x}},f_{0} )|,|S_{41} ({\boldsymbol{x}},f_{0} )|} \right\} + \\ + \beta \left[ {|S_{21} ({\boldsymbol{x}},f_{0} )| - |S_{31} ({\boldsymbol{x}},f_{0} )| - K_{P} } \right]^{2} \\ \end{gathered}$$
Objectives:	
Minimize matching and isolation at the target operating frequency *f*_0_	
Ensure the required power split *K*_*P*_ at *f*_0_	
Circuit: Compact rat-race coupler	$$\begin{gathered} U({\boldsymbol{x}}) = A({\boldsymbol{x}}) + \beta_{1} \left[ {\max \{ c({\boldsymbol{x}}) + 20,0\} /20} \right]^{2} \\ + \beta_{2} \left[ {|S_{21} ({\boldsymbol{x}},f_{0} )| - |S_{31} ({\boldsymbol{x}},f_{0} )|} \right]^{2} \\ \end{gathered}$$ where $$\begin{gathered} c({\boldsymbol{x}}) = \max \{ f \in [f_{0} - B,f_{0} + B]: \\ \max \{ |S_{11} ({\boldsymbol{x}},f)|,|S_{41} ({\boldsymbol{x}},f)|\} \\ \end{gathered}$$
Objectives:	
Minimize the footprint area *A*(***x***)	
Ensure equal power split at the operating frequency *f*_0_	
Maintain |*S*_11_|, |*S*_41_|≤ –20 dB over the bandwidth[*f*_0_ – *B*, *f*_0_ + *B*]	
Circuit: Triple-band power divider	$$\begin{gathered} U({\boldsymbol{x}}) = \max \{ \mathop {\max }\limits_{{k,l \in \{ 1,2,3\} }} |S_{kk} ({\boldsymbol{x}},f_{l} )|,\mathop {\max }\limits_{{l \in \{ 1,2,3\} }} |S_{23} ({\boldsymbol{x}},f_{l} )|\} \\ + \beta \sum\limits_{l = 1}^{3} {(|S_{21} ({\boldsymbol{x}},f_{l} )| - |S_{31} ({\boldsymbol{x}},f_{l} )|)^{2} } \\ \end{gathered}$$
Objectives:	
Ensure equal power split at the operating frequencies *f*_1_, *f*_2_, and *f*_3_	
Minimize input matching |*S*_11_|, output matching |*S*_22_| and |*S*_33_|, and isolation |*S*_23_| at *f*_1_, *f*_2_, and *f*_3_	

* The terms followed by *β* are penalty factors introduced to enforce the design constraints (e.g., required power split or matching/isolation bandwidth).

### Variable-fidelity modeling

The concept of multi-fidelity simulations was explored in microwave engineering for over two decades^102^. The primary purpose is to accelerate design procedures with evaluation accuracy traded-off for computational speedup^103^. Diverse ways of decreasing model resolution exist e.g., with simplified physics (equivalent networks instead of full-wave simulation), neglecting losses, assuming perfect electrical conductors to represent metals, or reducing computational domain. However, a versatile way is to reduce the mesh density of the structure under analysis^104^. This method also maintains a reasonable correlation between EM analysis results of different resolutions^105^. For most microstrip components, possible acceleration factors (simulation time of the high-resolution analysis versus low-resolution one) may be up to five or six, assuming time-domain simulation and the low-fidelity model correctly render essential details of the circuit output.

Depending on the application, the low-resolution model may need to be corrected to be reliably used for optimization (e.g., space mapping^106^) or modeling purposes (e.g., co-kriging^24^). It might be used uncorrected in some applications, e.g., pre-screening^107^. Most multi-fidelity procedures employ two levels of model resolution (low/coarse, high/fine). Recently, model management schemes have been proposed with model resolution adjusted continuously^108^.

**Fig. 1:**
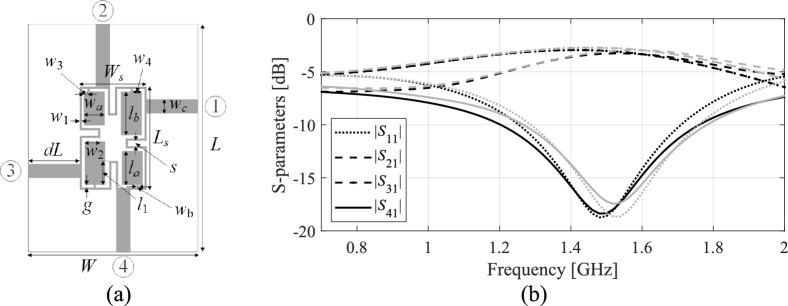
Multi-fidelity EM analysis: (**a**) a compact microstrip coupler, (**b**) *S*-parameters according to the low-resolution (gray) and high- resolution EM models (black). The high-fidelity model is evaluated in 210 s, whereas the low- resolution one in around 90 s.

Here, we employ two models: the high- and the low-resolution ones (***R***_*f*_(***x***) and ***R***_*c*_(***x***), respectively, cf. Figure 1). ***R***_*c*_(***x***) is employed to:

- Perform low-cost global sensitivity analysis (GSA), described in Section "Cost-effective global sensitivity analysis";
- Render the first (initial) metamodel (cf. Section "Cost-effective global sensitivity analysis");
- Conduct a machine-learning-based global search stage (cf. Section "Optimization process: AI-based global search phase").

The high-resolution model will perform final (gradient-based) parameter tuning (cf. Section "Optimization process: local parameter tuning"). As models of different resolutions exhibit good correlation, the GSA step may be realized using an uncorrected low-fidelity model. Similarly, surrogate modeling and global search can be executed using an uncorrected model because inaccuracies resulting from low-resolution EM analysis will be accommodated in the high-fidelity-based final tuning.

### Cost-effective global sensitivity analysis

The approach presented in this paper relies on behavioral models (surrogates). The major difficulties of data-driven modeling are linked to the curse of dimensionality, which causes a major expansion of the training dataset size required for building accurate metamodel with the number of independent variables. Other challenges arise due to the extensive ranges of geometry parameters the surrogate needs to cover and the nonlinearity of microwave component responses. These issues can be mitigated by reducing the parameter space dimensionality, which improves the relation between the model’s accuracy and the training point number.

Dimensionality reduction can be achieved by eliminating parameters having minor influence on the system characteristics. Some of available algorithmic tools involve variables screening approach (e.g., correlation coefficients: Pearson^109^ or partial^110^, Morris method^111^), and global sensitivity analysis approach (including Sobol^112^ and Jansen method^113^, or regression techniques^114^). Still, virtually all of these strategies are computationally expensive, i.e., sensitivity estimation requires training data sets of increased sizes. At the same time, eliminating individual parameters may not be the best option, especially for compact circuits featuring intricate parameter interactions regarding the degree of affecting the circuit scattering parameters. The aforementioned issues indicated the need for an alternative GSA approach that would be both fast and capable of identifying the most critical parameter space directions (regarding their effects on the system responses) rather than selecting individual variables to be eliminated. A procedure developed to satisfy these prerequisites was proposed in^115^.

#### Rapid global sensitivity analysis

In this study, we adopt low-cost rapid global sensitivity analysis (RGSA) proposed in^115^. The procedure of^115^ employs *N*_*s*_ random samples distributed in a uniform manner in the space *X* (using modified Latin Hypercube Sampling method^116^). Furthermore, the relocation matrix ***S*** is defined taking into account response variability data obtained from the samples’ nearest neighbors. More specifically, given the set of samples ***x***^(*k*)^, *k* = 1, …, *N*_*s*_, the data pair are created as follows. For each ***x***^(*k*)^, we find ***x***_*c*_^(*j*)^ = ***x***^(*j*min)^, where $$j_{\min } = \arg \mathop {\min }\limits_{\substack{ 1 \le j \le N_{s} \\ j \ne k } } \left\| {{\boldsymbol{x}}_{{}}^{(k)} - {\boldsymbol{x}}^{(j)} } \right\|$$. Further, we compute relocation vectors ***v***_*s*_^(*k*)^ = [***x***^(*k*)^ − ***x***_*c*_^(*k*)^]/||[***x***^(*k*)^ − ***x***_*c*_^(*k*)^|| and the corresponding response variabilities $r_{s}^{(k)} = ||{\boldsymbol{R}}_{f} ({\boldsymbol{x}}_{c}^{(k)} ) - {\boldsymbol{R}}_{f} ({\boldsymbol{x}}_{s}^{(k)} )||/||{\boldsymbol{x}}_{c}^{(k)} - {\boldsymbol{x}}_{s}^{(k)} ||$, for *k* = 1, …, *N*_*s*_. The *N*_*s*_ × *n* relocation matrix ***S*** is then defined as

4 $${\boldsymbol{S}} = \left[ {\begin{array}{*{20}c} {r_{s}^{(1)} ({\boldsymbol{v}}_{s}^{(1)} )^{T} } \\ \vdots \\ {r_{s}^{{(N_{s} )}} ({\boldsymbol{v}}_{s}^{{(N_{s} )}} )^{T} } \\ \end{array} } \right]$$

Once created, the matrix ***S*** undergoes the spectral analysis^117^. The eigenvectors of ***S*** are referred to as ***e***_*j*_ and correspond to the essential directions (i.e., those exerting the largest influence on system’s response), whereas the eigenvalues *λ*_*j*_, organized in descending order *λ*_1_ ≥ *λ*_2_ ≥ … ≥ *λ*_*n*_, quantify the mentioned significance.

RGSA aims to establish a reduced-dimensionality region as a domain for the globalized search (see Section "Optimization process: AI-based global search phase") and the data-driven metamodel constructed therein. The model’s domain is spanned by *N*_*d*_ < *n* most important vectors, where *N*_*d*_ ∈ {1, 2, …, *n*} corresponds to the smallest integer ensuring that

5 $$\frac{{\sqrt {\sum\nolimits_{j = 1}^{{N_{d} }} {\lambda_{j}^{2} } } }}{{\sqrt {\sum\nolimits_{j = 1}^{n} {\lambda_{j}^{2} } } }} \ge C_{\min }$$

*C*_min_ is a threshold value set to *C*_min_ = 0.9. Condition (5) is equivalent to selecting *N*_*d*_ so that the overall (relative) least-square response variability along ***e***_1_ through ***e***_*Nd*_ exceeds *C*_min_ (i.e., ninety percent for *C*_min_ = 0.9).

**Fig. 2:**
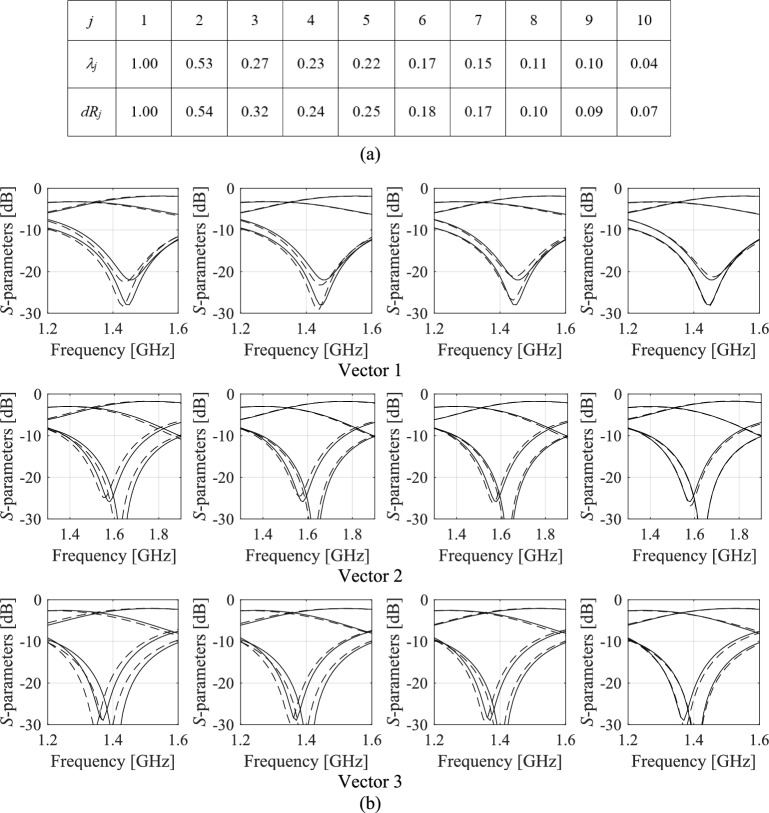
RGSA using the example of a miniaturized branch-line coupler from Fig. 1a: (**a**) scaled eigenvalues of ***S*** rendered via RGSA using 50 random samples and average *dR*_*j*_ (EM-evaluated variability factors) calculated using (5); (**b**) *S*-parameters at three selected parameter vectors (upper, centre, and lower row), and vectors shifted along the largest principal components (*k* = 1, …, 4 are shown from left to right)), ***x*** + *h****e***_*k*_ (we have *h* = 0.1, as this value enables obtaining a visible change of the circuit’s response for the most significant vectors ***e***_1_ and ***e***_2_) found through RGSA. Observe variability of the response subsides for successive *k*, showing that directions corresponding to the ensuing eigenvectors affect the circuit characteristics to a smaller extent.

Consider a microstrip coupler presented in Fig. 1a, whose design parameters, as well as lower and upper bunds are provided in Section "Verification circuits". For this example, RGSA^115^ has been executed using fifty random samples. The circuit frequency characteristics at three random parameter vectors, and vectors shifted along the eigenvectors, are shown in Fig. 2b. Note that, as predicted by eigenvalue analysis, the response variation is maximal for the first eigenvalue, and reduced for subsequent ones (cf. Figure 2b). It should be noted that while the response variations are reduced for the right-hand-side panels compared to the left-hand-side ones in Fig. 2b, one cannot expect strict monotonicity because of the random selection of the samples and the fact that the circuit response behavior depends on the particular location in the parameter space. Nonetheless, the overall trends agree with the predictions obtained through the global sensitivity analysis.

Response variability has been quantified through EM analysis of the circuit at *N*_*r*_ = 50 random vectors, ***x***_*r*_^(*k*)^, *k* = 1, …., *N*_*r*_, and the respective shifts ***x***_*r*_^(*k.l*)^ = ***x***_*r*_^(*k*)^ + *h****e***_*j*_, *j* = 1, …, *n*. In the following, ***R***_*c*_(***x***) will refer to the collective device’s responses, i.e., *S*_11_(***x***,*f*), *S*_21_(***x***,*f*), *S*_31_(***x***,*f*), and *S*_41_(***x***,*f*), assessed at frequencies *f* ∈ {*f*_1_, …, *f*_*m*_}, and evaluated at the low-resolution EM level. The variability factors are computed as

6 $$dR_{j} = \frac{1}{{N_{r} }}\sum\limits_{k = 1}^{{N_{r} }} {\left\| {{\boldsymbol{R}}_{c} ({\boldsymbol{x}}_{r}^{(k)} ) - {\boldsymbol{R}}_{c} ({\boldsymbol{x}}_{r}^{(k.j)} )} \right\|^{2} }$$

for *j* = 1, …, *n*. *dR*_*j*_ computed as in (6) corresponds to the average variability of the circuit’s characteristics along the direction ***e***_*j*_. Figure 2a demonstrates that normalized *dR*_*j*_ coincide with the normalized *λ*_*j*_, which confirms the significance of RGSA.

RGSA is developed to ensure computational efficiency and flexibility. It can be executed using a small number of observables, even though it is not as accurate as more expensive techniques^112,113^. On the other hand, this approach enables the determination of directions that are arbitrarily oriented with regard to the coordinate system axes, which makes it possible to explore parameter interactions rather than to eliminate individual variables. In other words, in our approach, the domain dimensionality is reduced, not the number of the design variables.

#### Dimensionality-reduced domain by RGSA

**Fig. 3:**
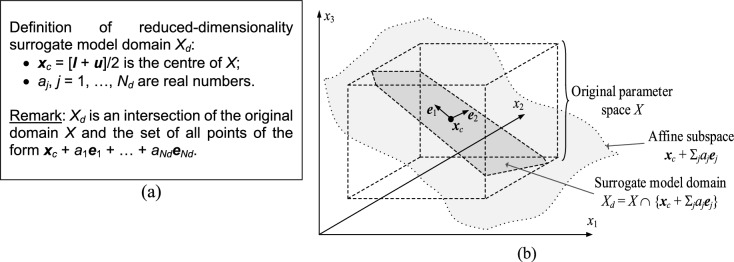
Reduced-dimensionality domain *X*_*d*_ using RGSA: (**a**) formal definition of the domain, (b) graphical illustration. Here, the initial design space has three dimensions, and *X*_*d*_ has two dimensions corresponding to the principal components ***e***_1_ and ***e***_2_. Observe that *X*_*d*_ represents an inter-section of *X* and ***x***_*c*_ + Σ_*j*=1,2 _*a*_*j*_***e***_*j*_ being an affine subspace.

As explained earlier, RGSA is used to find significant directions regarding their impact on circuit responses. These directions are now utilized for defining of the reduced set *X*_*d*_ being a domain during a global search. *X*_*d*_ is the region of the model’s validity (cf. Section "Optimization process: AI-based global search phase"). A definition of *X*_*d*_ has been detailed in Fig. 3a, whereas Fig. 3b contains a conceptual illustration.The set *X*_*d*_ is a result of intersecting of *X* and the affine sub-space spanned by the eigenvectors ***e***_1_ through ***e***_*Nd*_. The sub-space center is ***x***_*c*_, a geometrical mean of *X*. Reducing dimensionality profoundly affects a data-driven model’s predictive power and training data acquisition costs. While it is essentially undoable to construct a reliable surrogate within the original space *X*, reducing dimensionality to *N*_*d*_ being three to five makes it possible. Because *X*_*d*_ is spanned by the vectors that are essential from the standpoint of the circuit response variability, operating in this domain leads to meaningful results. Furthermore, the global search step will be complemented by local parameter adjustment (cf. Section "Optimization process: local parameter tuning"), compensating for potential inaccuracies caused by domain confinement.

### Optimization process: AI-based global search phase

According to the proposed methodology, optimization is organized into two steps. The first stage, global search, aims to find the high-quality subset of the space while operating in the dimensionality-reduced region *X*_*d*_. The second stage is a local tuning conducted in the original space *X*. Global optimization is carried out as ML procedure that involves kriging-based surrogate. The underlying infill criterion consists in the improvement of the surrogate-evaluated cost function. The core optimizer is PSO. To ensure computational efficiency, the ML stage is executed at the low-resolution EM model level.

#### Initial surrogate

The initial surrogate is rendered to launch the machine-learning process outlined in Section "Machine learning step". The modeling method is kriging interpolation^118^, whereas the region of interest is the RGSA-reduced domain *X*_*d*_ (see Section "Dimensionality-reduced domain by RGSA"). The number of training samples is set to *N*_*i*_*N*_*d*_ (*N*_*d*_ being the reduced domain dimensionality), and *N*_*i*_ = 20 in verification experiments of Section "Results". The training points ***x***_*B*_^(*k*)^, *k* = 1, …, *N*_*d*_*N*_*i*_, are distributed in *X*_*d*_ in a uniform fashion. First, we set up a temporary surrogate ***s***_*tmp*_(***x***) based on the set {***x***_*B*_^(*k*)^,***R***_*c*_(***x***_*B*_^(*k*)^)}_*k* = 1, …, *NiNd*_, with ***R***_*c*_(***x***_*B*_^(*k*)^) representing the relevant circuit responses evaluated through low-resolution EM analysis. Subsequently, the procedure for infill point generation is performed and ***s***_*tmp*_ is iteratively enhanced using the infill samples inserted via maximization of the modelled mean square error (MSE)

7 $${\boldsymbol{x}}_{B}^{{(N_{i} N_{d} + j)}} = \arg \mathop {\max }\limits_{{{\boldsymbol{x}} \in X_{d} }} MSE({\boldsymbol{s}}_{tmp} ({\boldsymbol{x}}))$$

for *j* = 1, 2, …. The training set is then extended as {***x***_*B*_^(*k*)^,***R***_*c*_(***x***_*B*_^(*k*)^)}_*k* = 1, …, *NdNi* + *j*_. The following termination criteria of model enhancement are used:

- Relative RMS error (assessed with the use of cross validation^119^) becomes smaller than *E*_max_ (control parameter of the algorithm), or
- The overall training dataset cardinality exceeds 2*N*_*i*_*N*_*d*_ (maximum computational budget).

The problem (7) is solved using PSO. The infill point generation procedure involves inserting the infill points in the location of maximum predicted MSE, thereby improving the metamodel’s accuracy in *X*_*d*_. The final version of ***s***_*tmp*_(***x***) will be assigned as the initial surrogate ***s***^(0)^(***x***).

#### Machine learning step

The first step of the optimization procedure constitutes a global search. It is executed inside the reduced domain *X*_*d*_ and implemented as an ML algorithm that involves kriging surrogates. The procedure starts by optimizing the surrogate model ***s***^(0)^, obtained as discussed in Section "Initial surrogate". Subsequent models, ***s***^(*i*)^, *i* = 1, 2, …, are identified based on the acquired simulation data. The surrogate is identified through maximum likelihood estimation, which is a simple local search task. The training time for the training dataset sizes used here is a few seconds (around one hundred samples). Therefore, the computational cost of this process is negligible.

The vector ***x***^(*i*+1)^ generated in the *i*th iteration, *i* = 0, 1, 2, …, is obtained through minimization of the function *U*_*S*_(***x***,***s***^(*i*)^(***x***)). *U*_*S*_ is analytically identical to the function *U* (see Section "EM-driven design of microwave circuits"); yet, for its evaluation ***s***^(*i*)^(***x***) is used instead of EM-simulated circuit responses. The vector ***x***^(*i*+1)^ is found as

8 $${\boldsymbol{x}}^{(i + 1)} = \arg \mathop {\min }\limits_{{{\boldsymbol{x}} \in X_{d} }} U_{S} ({\boldsymbol{x}},{\boldsymbol{s}}^{(i)} ({\boldsymbol{x}}))$$

In other words, the candidate solution is obtained as the best possible design according to the prediction of the surrogate model. In this study, PSO algorithm^120^ is utilized to solve the problem (8). We use the standard version of the PSO procedure without any modifications. It should be observed that virtually any nature-inspired optimization algorithm may be incorporated into our framework instead of PSO. This is because the computational cost of globally optimizing the surrogate is low. Consequently, optimization can be performed with a high number of objective function evaluations. In our numerical experiments, we perform surrogate optimization with the number of objective function evaluations set to 50,000. For such a large computational budget, the performance of various nature-inspired algorithms is similar. Thus, any search procedure of choice may be used to solve the problem (8).

In ML terms, the task (8) corresponds to the enhancement of the predicted objective function exploited as the infill criterion^121^. Thus, vectors ***x***^(*i*)^, *i* = 1, 2, …, become optimum design approximations, which (in addition to the corresponding EM simulation results) serve for surrogate model refinement. To be more precise, ***s***^(*i*)^(***x***) is obtained from $\{ \boldsymbol{x}_{\beta }^{(k)} \}_{{k = 1, \ldots ,2N_{i} N_{d} + i}} ,{\text{ with }}\boldsymbol{x}_{\beta }^{{(2N_{i} N_{d} + i)}} = \boldsymbol{x}^{(i)} {\text{ for }}i = 1,2, \ldots$.

The following termination conditions for the ML procedure are used: (i) $|\boldsymbol{x}^{(i + 1)} - \boldsymbol{x}^{(i)} | < \varepsilon$ (convergence in argument), or (ii) the cost function has not been improved was observed during the previous *N*_*no_improve*_ search cycles. Here, the control parameters *ε* and *N*_*no_improve*_ are set to *ε* = 10^–2^ and *N*_*no_improve*_ = 20.

### Optimization process: local parameter tuning

As explained earlier, conducting a global search in a reduced-dimensionality domain *X*_*d*_ is associated with profound computational benefits. However, even though *X*_*d*_ is defined to account for the primary response changes of the circuit, the actual optimum may be located outside this region. Furthermore, the low-resolution EM analysis for carrying out the ML process, which compromises the search process reliability. Accommodating these factors necessitates a supplementary optimization stage, which is final (local) refinement inside unreduced space *X*. Here, it is realized using accelerated gradient-based optimization. This part is realized at the level of the high-resolution EM analysis.

The local tuning algorithm is the trust-region (TR) algorithm^122^, which employs Taylor’s expansion model of the circuit outputs, which is optimized near the current design. Given the design problem stated as in (1), the TR algorithm operates by generating a series ***x***^(*i*)^, *l* = 0, 1, …, of approximations to x^*^, with ***x***^(0)^ being an initial design. The new vector ***x***^(*l*+1)^ is obtained by solving

9 $${\boldsymbol{x}}^{(l + 1)} = \arg \mathop {\min }\limits_{{{\boldsymbol{x}};\;||{\boldsymbol{x}} - {\boldsymbol{x}}^{(l)} || \le d^{(l)} }} U_{L} ({\boldsymbol{x}},{\boldsymbol{L}}_{{}}^{(l)} ({\boldsymbol{x}}))$$

where

10 $${\boldsymbol{L}}^{(l)} ({\boldsymbol{x}}) = {\boldsymbol{R}}({\mathbf{x}}^{(l)} ) + {\boldsymbol{J}}_{R} ({\boldsymbol{x}}^{(l)} ) \cdot ({\boldsymbol{x}} - {\boldsymbol{x}}^{(l)} )$$

is a first-order Taylor expansion of ***R*** at ***x***^(*l*)^. The Jacobian matrix ***J***_*R*_ is estimated using finite differentiation^122^. Observe, that the objective function *U*_*L*_^(*l*)^ (4) is analytically identical to *U*_*P*_ (cf. (2) and (3) but evaluated using ***L***^(*l*)^(***x***) rather than directly EM-simulated responses ***R***(***x***). The TR algorithm employs the gain ratio *r*, which is EM-evaluated versus linear-model predicted objective function improvement

11 $$r = \left[ {U_{P} ({\boldsymbol{x}}^{(l + 1)} ,F) - U_{P} ({\boldsymbol{x}}^{(l)} ,F)} \right]/\left[ {U_{L} ({\boldsymbol{x}}^{(l + 1)} ,{\boldsymbol{L}}^{(i)} ({\boldsymbol{x}}^{(l + 1)} ),F) - U_{L} ({\boldsymbol{x}}^{(l)} ,{\boldsymbol{L}}^{(l)} ({\boldsymbol{x}}^{(l)} ),F)} \right]$$

The trust region size *d*^(*l*)^ > 0 is adaptively adjusted based on *r*; *d*^(*l*+1)^ = *d*^(*l*)^*m*_*incr*_ if *r* > *r*_*incr*_, and *d*^(*l*+1)^ = *d*^(*l*)^/*m*_*decr*_ if *r* < *r*_*decr*_; standard control parameter values are *r*_*incr*_ = 0.75, *r*_*decr*_ = 0.25, *m*_*incr*_ = 1.5, *m*_*decr*_ = 2^122^. The new iteration point ***x***^(*i*+1)^ is accepted only if *r* > 0 (i.e., EM-evaluated objective function has been improved); otherwise, the iteration is repeated with reduced TR size. The algorithm termination is based on convergence in argument (||***x***^(*l*+1)^ – ***x***^(*l*)^||< *ε*_*TR*_) or sufficient reduction of the TR size (*d*^(*l*)^ ≤ *ε*_*TR*_); the termination threshold is set to *ε*_*TR*_ = 10^–3^.

During the initial iterations of the optimization process (in the final local refinement stage of the proposed framework), the Jacobian matrix is calculated using finite differentiation (FD)^123^, which is costly. The search region’s size is iteratively altered based on the estimated predictive power of Taylor’s model. Near convergence, the system’s response Jacobian is computed via the Broyden update^124,125^ rather than FD for cost reduction.

### Optimization framework complete procedure

The components discussed in Sections "Variable-fidelity modeling" through "Optimization process: local parameter tuning" are combined into a global optimization framework. As mentioned earlier, the initial stages (sensitivity analysis, surrogate model construction, ML-based global search) are executed with the low-resolution model ***R***_*c*_. In the final stage (local parameter refinement), high-fidelity simulations (i.e., model ***R***_*f*_) are used.

**Fig. 4:**
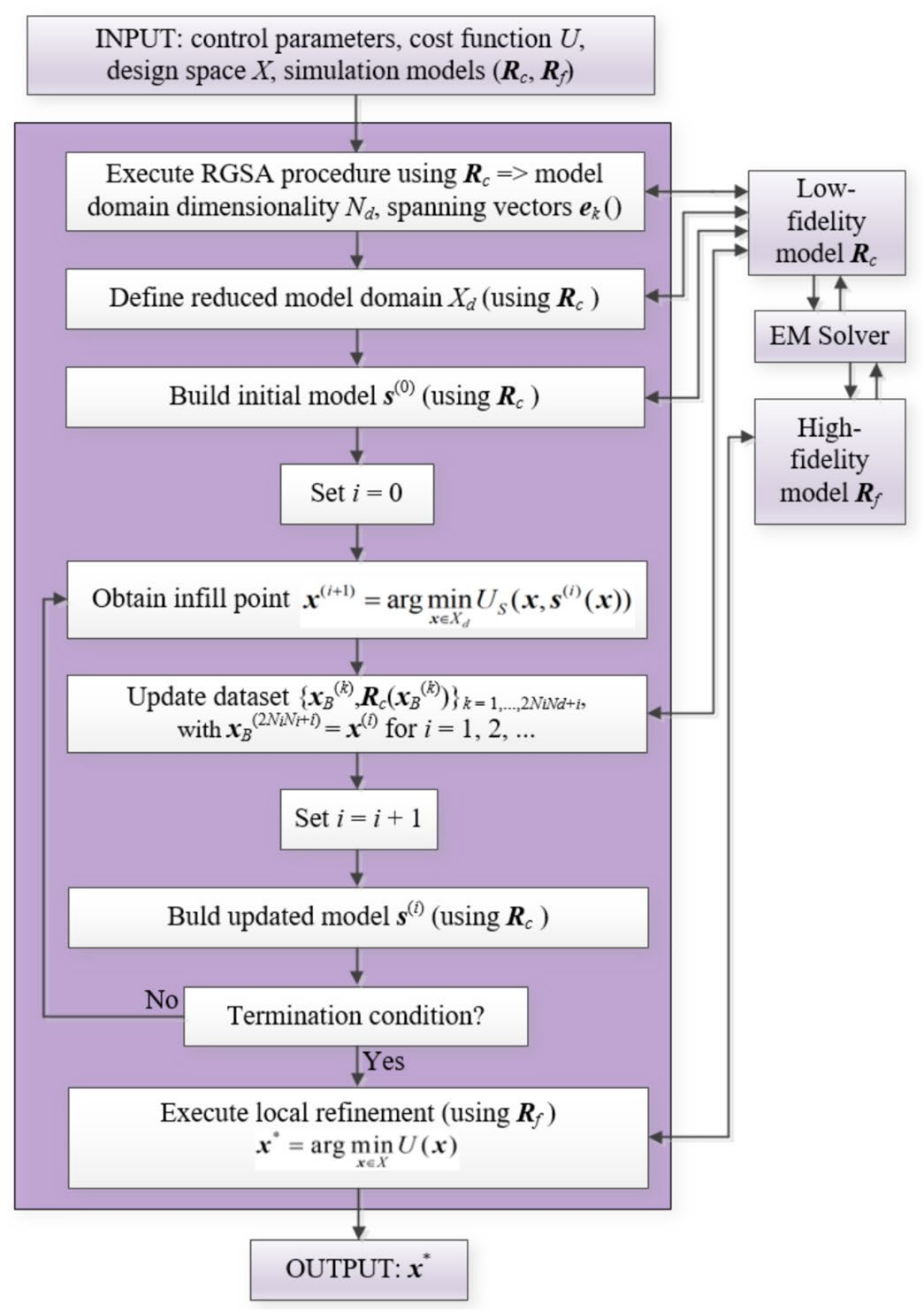
Proposed variable-resolution AI-based procedure for global microwave optimization: flow diagram.

The control parameters of the framework have been gathered in Table 3. Among these, three parameters adjust the optimization process resolution (*ε*, *N*_*no_improve*_, *ε*_*TR*_). The remaining three, *N*_*r*_, *N*_*i*_, and *E*_max_, are used to determine the computational budget of the RGSA procedure and initial surrogate model construction, and its required predictive power. None of these parameters is critical. In particular, RGSA estimation results from averaging the circuit response variability over the entire parameter space. Consequently, its outcome is only affected by the number of involved observables to a small extent. Furthermore, the dependability of the initial surrogate (controlled by both *N*_*i*_ and *E*_max_) is not critical either, as the model is refined in the ML process anyway. To demonstrate that, identical algorithm setup is employed in all numerical experiments of Section "Results" (as shown in the Table 3). Whereas Fig. 4 presents the flow diagram of the introduced variable-resolution optimization framework. The main stages are fast sensitivity analysis, downsizing the domain dimensionality and domain definition, initial surrogate model construction, ML-based global optimization, and final gradient-based tuning. Low-resolution EM analysis is utilized in all stages but the last one.

**Table 3:** Control parameters of the proposed variable-resolution AI-based procedure for global microwave optimization.

Parameter/default value	Meaning
*N*_*r*_ = 50	Number of random observables for RGSA
*N*_*i*_ = 20	Multiplier for the number of uniformly-distributed data samples for initial surrogate model construction. Total number of samples is *N*_*i*_*N*_*d*_, where *N*_*d*_ denotes dimensionality of the reduced domain *X*_*d*_
*E*_max_ = 20%	Maximum value of relative RMS error of the initial surrogate model
*ε* = 10^–2^	Termination threshold for convergence in argument
*N*_*no_improve*_ = 10	Termination threshold for no objective function value improvement
*ε*_*TR*_ = 10^–3^	Termination threshold for local parameter tuning stage

It should be emphasized that both dimensionality reduction and multi-fidelity simulations play essential roles in accelerating the optimization process. Dimensionality reduction enables a cost-efficient rendition of a reliable surrogate model, which improves the performance and reduces the expenses of the global search stage. By conducting this step at the level of a low-fidelity EM model, further efficacy enhancement is possible. At the same time, dimensionality reduction (and the associated improvement of the surrogate model accuracy) enables the generation of a good-quality initial design for the final tuning, reducing the latter’s cost.

## Results

The procedure of Section "Variable-resolution microwave optimization by dimensionality-reduced surrogates and artificial intelligence-based methods" is demonstrated here based on four microstrip circuits introduced in Section "Verification circuits". Our methodology is compared in Section "Setup and results" to several benchmark techniques that include a population-based metaheuristic, standard gradient-based optimization, and ML routines operating in the original and the reduced space (both exclusively using high-resolution EM simulations). The results obtained for multiple algorithm runs are discussed Section "Result summary and discussion", which also summarizes the overall performance of the framework.

### Verification circuits

**Fig. 5:**
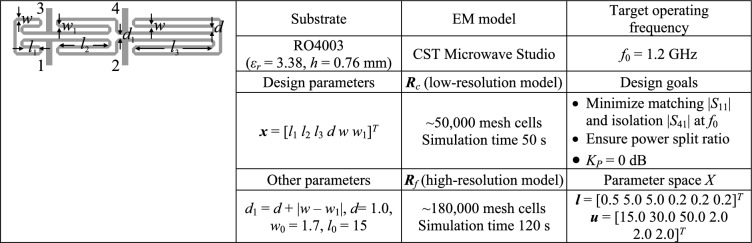
Verification example I^126^: (**a**) circuit geometry, (**b**) essential data.

**Fig. 6:**
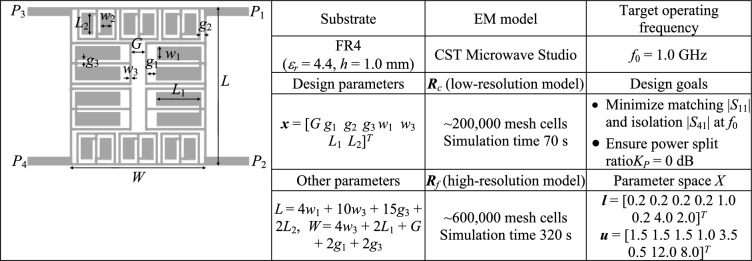
Verification example II^127^: (**a**) circuit geometry, (**b**) essential data.

**Fig. 7:**
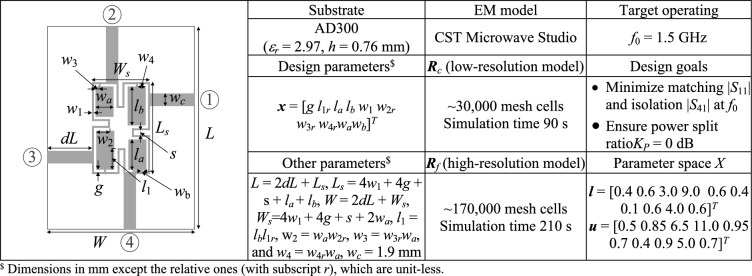
Verification example III^128^: (**a**) circuit geometry, (**b**) essential data.

**Fig. 8:**
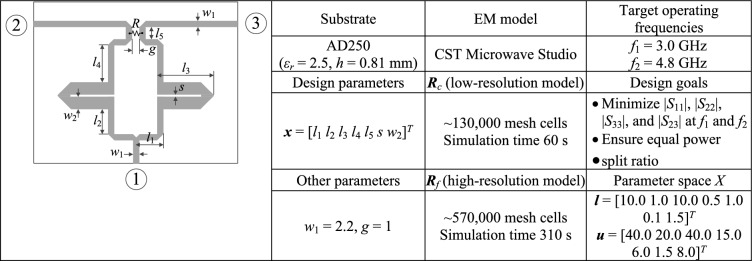
Verification example IV^129^: (**a**) circuit geometry, (**b**) essential data.

In pursuit of meaningful performance verification of our algorithm, we utilize it to carry out global parameter adjustment of four microstrip circuits, which include (i) a compact rat-race coupler (RRC) with folded transmission lines (Circuit I)^126^; (ii) a compact branch-line coupler with microstrip cells (Circuit II)^127^; (c) a miniaturized branch-line coupler (Circuit III)^128^; and (iv) a dual-band power divider (Circuit IV)^129^. The circuit architectures as well as important data concerning substrate material, design variables, parameter space *X*, and design specifications have been presented in Figs. 5, 6, 7 and 8.

**Fig. 9:**
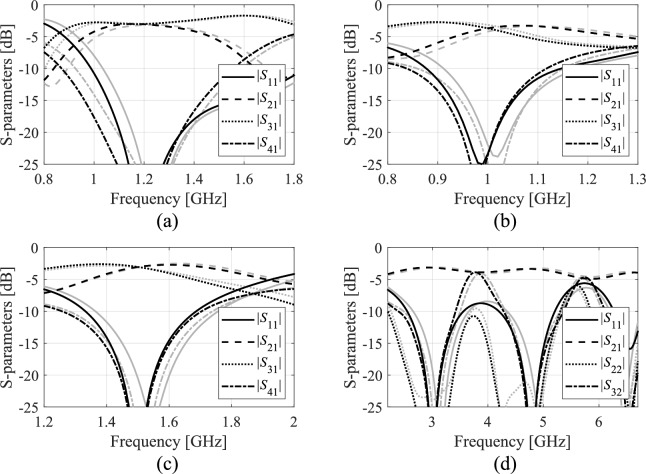
High-fidelity (black) and low-fidelity (gray) EM models at selected designs: (**a**) Circuit I, (**b**) Circuit II, (**c**) Circuit III, (**d**) Circuit IV). The main discrepancy between the high- and low-fidelity models are frequency shifts and slight level differences.

Full-wave simulation models are evaluated with the use of CST Microwave Studio^130^. The low-resolution model ***R***_*c*_ is determined by diminishing the antenna’s discretization density as much as possible so that the model can still account for the essential features of the circuit responses. The configuration of fine model ***R***_*f*_ is assessed using the grid convergence approach (such that additional increases in resolution do not result in noticeable changes to the circuit characteristics). The computational savings resulting from incorporating variable-resolution models are contingent upon the simulation duration ratio between ***R***_*f*_ and ***R***_*c*_, which is 2.4, 4.6, 2.1, and 5.2 for Circuit I, II, III, and IV, respectively. Needless to say, a higher ratio implies better computational efficiency. For additional explanation, Fig. 9 presents the responses of each verification circuit at selected designs evaluated using low- and high-resolution EM analysis. As indicated in the pictures, the main discrepancy between the low- and high-fidelity models is frequency shifts and slight differences in response levels. Due to these misalignments, the low-fidelity model is not suitable to be the only circuit representation in the optimization process. However, the final tuning step carried out using the high-fidelity simulations accounts for these inaccuracies and ensures reliability.

**Fig. 10:**
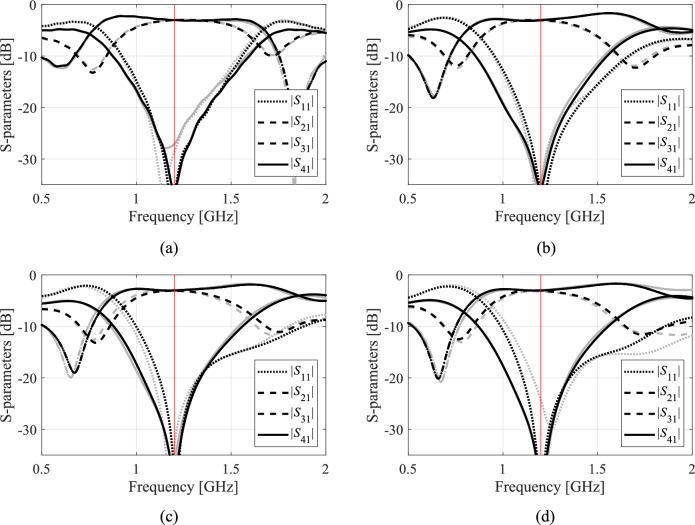
Circuit I: frequency characteristics for the selected solution geometry parameters obtained using the presented ML algorithm: (**a**)–(**d**) solution 1 through 4. Grey lines indicate the responses at the starting design found during the global search. The circuit characteristics at the final design are shown using black lines. The operating frequency equal 1.2 GHz is indicated via vertical line.

**Fig. 11:**
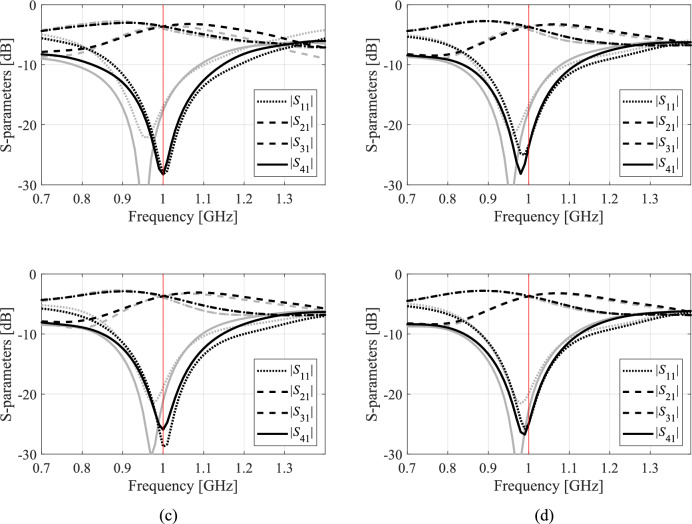
Circuit II: frequency characteristics for the selected solution geometry parameters obtained using the presented ML algorithm: (**a**)–(**d**) solution 1 through 4. Grey lines indicate the responses at the starting design found during the global search. The circuit characteristics at the final design are shown using black lines. The operating frequency equal 1.0 GHz is indicated via vertical line.

**Fig. 12:**
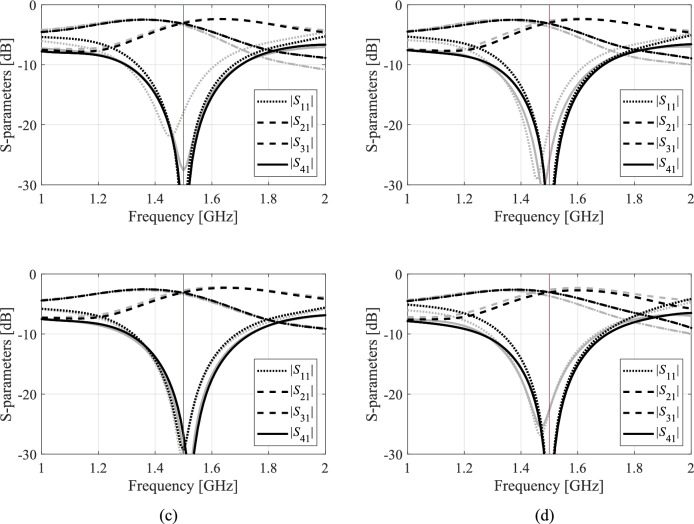
Circuit III: frequency characteristics for the selected solution geometry parameters obtained using the presented ML algorithm: (**a**)–(**d**) solution 1 through 4. Grey lines indicate the responses at the starting design found during the global search. The circuit characteristics at the final design are shown using black lines. The operating frequency equal 1.5 GHz is indicated via vertical line.

**Fig. 13:**
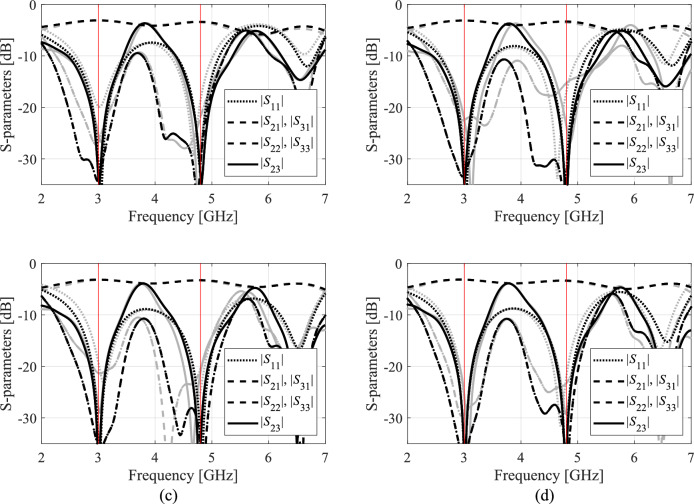
Circuit IV: frequency characteristics for the selected solution geometry parameters obtained using the presented ML algorithm: (**a**)–(**d**) solution 1 through 4. Grey lines indicate the responses at the starting design found during the global search. The circuit characteristics at the final design are shown using black lines. The operating frequencies equal 3.0 GHz and 4.8 GHz are indicated via vertical lines.

A closer look at data presented in Figs. 5, 6, 7 and 8 reveals that the optimization tasks are challenging. On the one hand, this is due to the number of variables (between six and ten, depending on the circuit), broad ranges of parameters (the upper versus lower bound ratios are 13, 5, 2, and 12 for Circuit I, II, III, and IV, respectively), the need for processing four circuit characteristics (e.g., *S*_11_, *S*_21_,* S*_31_, and *S*_41_ in the case of couplers) over wide frequency spectra, but also nonlinearity of the frequency responses (cf. Figures 10, 11, 12 and 13).

### Setup and results

Table 3 provides information about the configuration of our algorithm. For all circuits the preset values of the control parameters provided in Table 3 are used. We also use four benchmark techniques, which are summarized in Table 4. The first of these (Algorithm I) is the particle swarm optimizer (PSO)^120^. It is included as a commonly adopted nature-inspired procedure. Two options differing in computational budget are considered: 500 (Option I) and 1,000 (Option II) cost function evaluations. The cited numbers may be moderate for population-based algorithms, yet, they are substantial from the perspective of the CPU time (two to four days of a running time). Both versions of Algorithm I utilize high-resolution EM simulation models.

**Table 4:** Benchmark algorithms.

Algorithm	Algorithm type	Setup
This work	RGSA-based surrogate-assisted machine-learning framework with dimensionality reduction	Control parameters: *N*_*r*_ = 50, *N*_*i*_ = 20, *E*_max_ = 20%, *ε* = 10^–2^, *N*_*no_improve*_ = 20, *ε*_*TR*_ = 10^–3^ (the meaning of parameter has been explained in Table 3)
I	Particle swarm optimizer (PSO)	Swarm size *N* = 10, standard control parameters (*χ* = 0.73, *c*_1_ = *c*_2_ = 2.05); number of iterations set to 50 (version I) and 100 (version II)
II	Trust-region gradient based optimizer	Random initial design, response gradients estimated using finite differentiation, termination criteria based on convergence in argument and reduction of the trust region size^121^
III	Machine-learning procedure	Algorithm setup:
		Initial surrogate set up to ensure relative RMS error not higher than 20% with the maximum number of training samples equal to 400;
		Algorithm operates in the original parameter space (no dimensionality reduction);
		Infill criterion: minimization of the predicted objective function
IV	Machine-learning procedure	Algorithm setup:
		The method is the same as the proposed one; however, the algorithm operates at the level of high-resolution EM models;
		Control parameters: default values as in Table 3

The second procedure (Algorithm II) is a local method with numerical derivatives initiated from randomly selected initial point. It is a conventional TR algorithm (cf. Section "Optimization process: local parameter tuning"). It is included to corroborate multimodality of the test problems. The third compared method (Algorithm III) is an ML framework operating in the design variables space *X*, which involves kriging metamodels and the predicted objective function improvement to generate the candidate designs. The purpose of employing Algorithm II is to verify the benefits coming from dimensionality reduction. The last benchmark technique (Algorithm IV) is a ML algorithm operating in a reduced-dimensionality domain but exclusively using high-resolution EM models. It is included in the comparison pool to demonstrate the potential merits of including variable-resolution EM simulations in the search process.

The results obtained for all Circuits are summarized in Tables 5, 6, 7, and 8, respectively. Ten runs of each algorithm were performed, with the tables reporting the mean values of performance figures, including the objective function at the optimized design and the computational expenses incurred by the search routine (i.e., the number of high-resolution EM simulations). Another reported figure is the number of algorithm executions that produced acceptable results (success rate) concerning the agreement between the circuit operating frequencies and the specified targets. The circuit outputs for the representative runs of our procedure have been shown in Figs. 10, 11, 12 and 13. The figures show the frequency characteristics at the designs produced during global search and the designs generated by means of the final tuning.

**Table 5:** Circuit I: Results and benchmarking.

Optimization procedure		Performance figure		
		Average cost function [dB]	Computational cost^$^	Success rate^#^
I	PSO (50 iterations)	− 24.8	500	9/10
	PSO (100 iterations)	− 34.0	1,000	10/10
II	TR routine	− 18.7	102.8	6/10
III	ML executed in the design space *X* of full dimensionality	− 34.1	435.7	10/10
IV	ML executed in the reduced space *X*_*d*_; high-resolution model only	− 38.6	155.0	10/10
Proposed algorithm		− 38.1	80.0	10/10

^$^The cost is expressed in terms of the the equivalent number of high-fidelity EM simulations of the circuit.

^#^Number of algorithm runs for which the operating frequencies were allocated near the target frequency.

**Table 6:** Circuit II: Results and benchmarking.

Optimization procedure		Performance figure		
		Average cost function [dB]	Computational cost^$^	Success rate^#^
I	PSO (50 iterations)	− 20.8	500	9/10
	PSO (100 iterations)	− 22.2	1,000	9/10
II	TR routine	− 7.5	49.0	5/10
III	ML executed in the design space *X* of full dimensionality	− 26.2	449.8	10/10
IV	ML executed in the reduced space *X*_*d*_; high-resolution model only	− 24.5	183.6	10/10
Proposed algorithm		− 25.0	66.0	10/10

^$^The cost is expressed in terms of the the equivalent number of high-fidelity EM simulations of the circuit.

^#^Number of algorithm runs for which the operating frequencies were allocated near the target frequency.

**Table 7:** Circuit III: Results and benchmarking.

Optimization procedure		Performance figure		
		Average cost function[dB]	Computational cost^$^	Success rate^#^
I	PSO (50 iterations)	− 25.2	500	9/10
	PSO (100 iterations)	− 29.1	1,000	9/10
II	TR routine	− 10.7	57.4	8/10
III	ML executed in the design space *X* of full dimensionality	− 30.2	238.4	10/10
IV	ML executed in the reduced space *X*_*d*_; high-resolution model only	− 25.7	165.4	10/10
Proposed algorithm		− 35.7	122.2	10/10

^$^The cost is expressed in terms of the the equivalent number of high-fidelity EM simulations of the circuit.

^#^Number of algorithm runs for which the operating frequencies were allocated near the target frequency.

**Table 8:** Circuit IV: Results and benchmarking.

Optimization procedure		Performance figure		
		Average cost function [dB]	Computational cost^$^	Success rate^#^
I	PSO (50 iterations)	− 23.7	500	9/10
	PSO (100 iterations)	− 36.2	1,000	10/10
II	TR routine	48.3	68.7	5/10
III	ML executed in the design space *X* of full dimensionality	− 45.6	433.6	10/10
IV	ML executed in the reduced space *X*_*d*_; high-resolution model only	− 42.5	282.2	10/10
Proposed algorithm		− 38.5	100.7	10/10

^$^The cost is expressed in terms of the the equivalent number of high-fidelity EM simulations of the circuit.

^#^Number of algorithm runs for which the operating frequencies were allocated near the target frequency.

### Result summary and discussion

Here, we analyze the numerical results and summarize the presented procedure’s performance. Furthermore, comparison with the benchmark procedures (Algorithms I through IV) is also provided. A crucial part of the following considerations constitute performance factors reported in Tables 5, 6, 7 and 8.

*Reliability and global search capability*. The success rate mentioned earlier is the metric assumed to evaluate the ability to carry out the global search. The outcome of Algorithm II (TR started form multiple initial points) corroborates the multimodality of the considered tasks as the success runs of this method were equal to 6, 5, 8 and 5 (out of ten algorithm runs) for Circuit I, II, III, and IV, respectively. In particular, it demonstrates that the outcome is highly dependent on the starting point (chosen randomly in the case of Algorithm II). Under these circumstances, our method shows a perfect success rate 10/10 in the case of all considered structures. ML-based procedures (Algorithms III and IV) deliver equally good results, which is to be expected given that all these methods share similar working principles. However, the computational efficiency of the benchmark techniques is considerably worse (detailed analysis thereof will be provided later). Nature-inspired optimization, here, PSO, does not perform as well. Its average success rate is 9/10 for the computational budget of 500 objective function evaluations and 9.5/10 for the budget of 1,000 calls. These numbers indicate that—for the considered verification cases—PSO requires a budget of at least 2,000 objective function evaluations to yield consistent results. As mentioned before, the setup assumed here was intentional to avoid excessive running times, which are significant anyway (from two to almost four CPU days per algorithm run).

*Design quality*. The outcome of the presented algorithm is excellent in all considered cases. The mean cost function values are significantly improved over those delivered by Algorithm II, and comparable to Algorithms III and IV (note that at the objective function levels of below –30 dB the differences of a few dB are not meaningful). The latter demonstrate that the acceleration mechanisms implemented in our framework are not detrimental to the reliability of the search process. When compared with Algorithm I (PSO), the average objective function values rendered by the proposed technique are better by a large margin. At the same time, a noticeable improvement can be observed between the two PSO versions: increasing the budget from 500 to 1,000 enhances the objective function value by almost 7 dB on the average, which confirms what was already stated in the previous paragraph: ensuring consistency of nature-inspired optimization necessitates significantly larger computational budgets.

*Cost efficacy*. The CPU cost entailed by the proposed methodology is exceptionally low. The mean cost, represented by the number of high-fidelity EM simulations, corresponds to only about ninety per algorithm run. This is comparable with the expenses typical for local search (an average of about seventy across the verification set of Circuit I through IV). The savings over Algorithm I (PSO) are 91 percent, 73 percent over Algorithm III, and 51 percent with respect to Algorithm IV. These figures indicate that our framework is about eleven, four, and over two times faster than PSO, Algorithm III, and Algorithm IV. This level of acceleration does not compromise the dependability of the optimization process. At this point, it should be emphasized that while incorporating the concept of ML contributes to the excellent operation of the presented methodology regarding the design quality, the cost efficiency is enhanced mainly by dimensionality reduction and utilization of variable-resolution models: each of these reduces the operating cost by approximately fifty percent. Observe that the verification Circuits I through IV are described by the following numbers of the geometry parameters 6, 8, 10, and 8, respectively. At the same time, the number of high-fidelity EM simulations required to find the globally optimal designs by the proposed algorithm is equal to 80, 66, 122, and 100. Thus, the relationship between the computational expenses and the number of variables is 11.2 on average. This permits assessing that the computational cost of our procedure is slightly over ten times higher than the number of design variables. This is remarkably low, given that our approach permits obtaining globally optimal solutions. As a matter of fact, this means that the cost of our procedure is only 50% higher than that of the local algorithms and nearly five times lower than that of machine-learning-based optimization in the standard domain. Moreover, the cost of our approach is around one-tenth of the cost of PSO optimizer (Algorithm I, version 2).

Based on the observations formulated above, it can be concluded that the framework suggested here is highly competitive over the considered benchmark techniques. On the one hand, it exhibits the ability to identify global optimum, perfect success rate (i.e., satisfactory results obtained for all algorithm runs), and consistent operation over the entire set of verification circuits. On the other hand, its computational efficiency is remarkably good and (almost) comparable with local search. Further, through comparisons with Algorithms III and IV, it has been corroborated that both multi-fidelity EM simulations and dimensionality reduction play crucial roles in enhancing cost efficiency, achieved without jeopardizing reliability and the quality of the optimal designs found by our routine. From a design utility perspective, another benefit of our methodology is a minimum number of user-defined parameters. Furthermore, their values are not critical due to the implemented self-adjustment mechanisms (e.g., improvement of the surrogate model predictive power through sequential sampling). An identical setup for all four verification circuits demonstrated no need for tuning the algorithm.

## Conclusion

This research aimed to introduce a high-performance technique for globalized parametric optimization of passive microwave circuits. Our method involves several algorithmic components devised to enhance accuracy and cost efficiency. Optimization relies on machine learning and local parameter tuning. The ML procedure operates in a reduced-dimensionality domain, which facilitates the construction of an accurate behavioral model employed as a predictor. Domain determination utilizes a set of vectors responsible for significant variations of the system’s outputs, determined using devised in this study fast global sensitivity analysis (RGSA). For further speedup, low-resolution EM analysis is employed for carrying out both RGSA, surrogate model rendition, and ML. The final gradient-based tuning operates in a full-dimensionality space to ensure reliability and employs the high-resolution EM model.

Extensive numerical experiments involving four microstrip circuits demonstrate superior and consistent performance of the presented framework, as well as it global search capability. Benchmarking against nature-inspired optimization, gradient-based routines, and machine-learning-based procedures operating in the full-dimensionality space indicate that our methodology is competitive regarding the design quality and the cost efficacy of the optimization procedure. The latter equals only ninety high-resolution EM analyses of the circuit on average, so it is approximately equal to the cost of a local search. This feature is a joint effect of dimensionality reduction and incorporating variable-resolution simulations. Dimensionality reduction renders about fifty percent savings, whereas employing low-resolution EM simulations contributes to an additional fifty percent cost reduction. The total (average) savings regarding the baseline ML reach 73 percent. Other important benefits coming from the employment of our approach are versatility (no assumptions are being made concerning the device at hand or its frequency responses), simple setup (owing to the limited number of control parameters), and simple implementation implied by a modular arrangement of individual building blocks. These characteristics make the proposed framework an viable alternative to the existent global optimization methodologies.

## Data Availability

Data availability: The datasets generated during and/or analysed during the current study are available from the corresponding author on reasonable request. Contact person: anna.dabrowska@pg.edu.pl.
